# Determinants for Acceptance of COVID-19 Vaccine in Nigeria

**DOI:** 10.7759/cureus.19801

**Published:** 2021-11-22

**Authors:** Ugochukwu A Eze, Kingsley I Ndoh, Babalola A Ibisola, Chinemerem D Onwuliri, Adenekan Osiyemi, Nnamdi Ude, Amalachukwu A Chime, Eric O Ogbor, Adegboyega O Alao, Ashiru Abdullahi

**Affiliations:** 1 Department of Ophthalmology, Federal Medical Centre, Asaba, NGA; 2 Department of Public Health, University of Suffolk, Ipswich, GBR; 3 Health Economics Program, Department of Economics, Kaduna State University, Kaduna, NGA; 4 Department of Global Health, University of Washington, Seattle, USA; 5 Department of Family Medicine, University College Hospital, Ibadan, NGA; 6 Department of Community Medicine, University of Nigeria Teaching Hospital, Enugu, NGA; 7 Department of Ophthalmology, University of Nigeria Teaching Hospital, Enugu, NGA; 8 Department of Ophthalmology, Jos University Teaching Hospital, Jos, NGA; 9 Department of Ophthalmology, Federal Medical Centre, Katsina, NGA

**Keywords:** nigeria, determinants, covid-19, adults, acceptance

## Abstract

Background: The coronavirus disease 2019 (COVID-19) pandemic heralded an unprecedented race to the development of several vaccine candidates at record speeds never seen in global health. Within nine months, Pfizer-BioNTech’s COVID-19 vaccine was approved by the United States FDA. Unfortunately, while these advances were ongoing, there was a burgeoning epidemic of disinformation about the virus and the vaccines that affected the willingness of people, especially minority groups, to get vaccinated. In Nigeria, this wave of vaccine hesitancy was happening against the backdrop of landmark pharmaceutical litigations such as the 2007 Pfizer trovafloxacin lawsuit in the country.

Aim: To assess the determinants of the COVID-19 vaccine's acceptability among Nigerians.

Materials and methods: Following ethical approval, a population-based cross-sectional study was conducted from November 2020 to January 2021 using an adapted pretested, self-administered questionnaire originally designed by Amyn Malik and colleagues who conducted a similar study at Yale University School of Public Health. The participants were recruited through simple random sampling using a list of community and corporate sites obtained from Google Maps in the three regional zones of Nigeria (north, east, and west) in diverse occupational and residential settings. Information obtained includes socio-demographics, medical history related to COVID-19, level of knowledge, risk perception, and attitudes toward COVID-19 and the vaccines. Descriptive and inferential statistics were done, and results were summarized into percentages and associations. The level of statistical significance was set at a p-value of <0.05. Using the open EpiR package (Emory), we determined a minimum of 340 participants for a statistical power of 80%.

Results: A total of 358 responses were obtained out of the 120 questionnaires distributed in each of the three regions, of which 189 (53%) were females. The mean age of respondents was 32 years (±11.2 SD). About 75% of the participants had at least a college education. The majority (66.2%) of the participants were willing to accept the approved vaccine. The mean risk perception score for COVID-19 was 5.1 (±2.2 SD) out of 10, while the mean COVID-19 symptom knowledge score was 8.6 (±4.1 SD) out of 19. Variables such as being male, identifying as Christian, Hausa ethnicity, and living in northern Nigeria had a statistically significant relationship with the willingness to get vaccinated.

Conclusion: Over 60% of Nigerians are willing to take the COVID-19 vaccines if recommended by health workers. We found male gender, religion, ethnicity, and geographical location to positively influence the willingness of Nigerians to get vaccinated against COVID-19. Health workers should be supported to go beyond the confines of the hospital to educate the general public in schools, marketplaces, churches, and corporate organizations on the efficacy and safety of the approved vaccines.

## Introduction

The coronavirus disease 2019 (COVID-19) was first reported in the Wuhan province of China in December 2019 before spreading rapidly throughout the globe. It was first reported in Nigeria on February 27, 2020. Around the same period, experts projected that Nigeria will likely become the pandemic’s epicenter in Africa [1]. Contrary to these projections, the disease ran a relatively less severe course in Nigeria and in most countries south of latitude 350 north, within which the whole of Sub-Saharan Africa falls [1]. As of March 2020, the World Health Organization published a case fatality rate (CFR) of 3-4%. In the West African sub-region, the average CFR is 3.9% [2]. In February 2021, Suleiman et al. estimated the CFR in Nigeria using polynomial regression models to be 3.0% (95% CI: 2.23-3.42%) [3]. This estimate would have been lower if there was widespread testing. Despite speculations that Africa would be the worst hit by the pandemic due to its weak healthcare systems and relatively higher population density, the predicted catastrophe is yet to happen [4]. As of September 24th, 2021, about 3 million COVID-19 tests (1.5% of the population) have been conducted with about 6% confirmed to be positive. Similarly, COVID-19 vaccinations have remained relatively low as only 1.58 million (0.8%) people have been fully vaccinated as of September 24th, 2021 [5].

Unfortunately, despite the daily exponential increases in recorded cases in Nigeria, there is a growing body of anecdotal evidence that many Nigerians do not believe in the existence of the pandemic. This is due in part to low trust levels in the government and a plethora of conspiracy theories surrounding the virus and vaccines by influential Nigerian political and religious leaders [6]. The surprisingly low CFR may have also contributed to the attitude of Nigerians and Africa at large towards the existence of this virus.

A vaccine is a live or attenuated antigen used to stimulate antibody production to provide immunity against pathogens like bacteria and viruses. Vaccination is one of the most potent preventive strategies against infectious diseases [7]. However, acceptance and coverage are vital determinants for a successful vaccination program [8]. With the approval and rollout of some efficacious and safe vaccines against severe acute respiratory syndrome coronavirus 2 (SARS-Cov-2), there has been an attendant epidemic of misleading information about the approved COVID-19 vaccines. This misinformation is not limited to Nigeria alone. Several media reports suggest that distrust in the United States and Europe has become a major obstacle to wider vaccine coverage [9-11]. Malik et al. at Yale University found that only 67% of the Americans they surveyed were willing to be vaccinated against COVID-19 before any vaccine was approved. This proportion has however improved since then [8,12]. Surveys conducted by GeoPoll in some African countries show that vaccine hesitancy decreases as more vaccines are rolled out [13]. As of September 2021, only about 1.5% of Nigerians have been vaccinated [6]. There are mixed feelings about the facts established on the evolution of the pandemic as well as with the vaccine’s efficacy and safety [14]. Despite these reactions, an online survey in Nigeria that was conducted just before the first-ever vaccine was approved reported that 58.2% of respondents would receive the vaccine once available while 19.2% and 22.6%, respectively, were unwilling or indecisive [15].

The purpose of our study is to assess the degree of acceptance of the approved COVID-19 vaccines among Nigerians, and the factors that influence the decisions to get vaccinated. The findings of our study are intended to support health policymakers, donor agencies, and other critical stakeholders to implement evidence-based strategies that are likely to be effective in the reduction of vaccine hesitancy and to ultimately increase vaccine uptake as more doses become available in Nigeria.

## Materials and methods

Study area

Nigeria is on the western coast of Africa. It is the most populous country in Africa, with an estimated population of about 206 million people [16]. It has 36 states, which are broadly divided into three ethnic-regional blocs: Eastern, Northern, and Western Nigeria.

Study design and participants

We conducted a cross-sectional study across Eastern, Northern, and Western Nigeria over a period of three months from November 2020 to January 2021. We used the open EpiR package (Emory) to generate the required minimum sample size of 340 for a population of one million and above [17].

We recruited 360 participants in total, 120 from each zone. Our recruitment was targeted at people in residential areas, corporate organizations, schools, recreational areas, faith-based institutions, and marketplaces. We did this by using Google Maps to list the aforementioned sites in two major states of each zone (north, west, and east). We then organized the list on Microsoft Excel version 16.54 (Microsoft Corp., Redmond, WA) and used the *Rand()* function to randomize each site list, and then we selected two sites for the recruitment. For the institutions, we contacted the human resources or desk offices to grant permission to visit and interview eligible participants in these sites. For the residential areas, we repeated the same Microsoft Excel process to randomize and select local government areas (counties) in the two states in each zone and residences to visit. Study participants who were 18 years and older at the time of data collection were eligible to be included in the study. Our exclusion criteria were people who were below 18 years and those who did not fully understand the reason for the study even after our explanations.

Study tool

Our data collection instrument was a pretested, self-administered questionnaire formulated from a similar study in the United States adapted to suit the Nigerian setting [9]. The questionnaire was divided into five sections comprising participants' socio-demographics, medical history, knowledge of COVID-19, risk perception on contracting COVID-19, and personal and behavioral attitudes to the approved COVID-19 vaccines.

Scales of measurement

Three multiple-choice questions (totaling 10 options) were asked to assess respondents' knowledge about the transmission of the COVID-19 virus. The scale was called the viral transmission knowledge scale (Cronbach's alpha = 0.62). A maximum score of 10 was achievable. These responses had reverse meaning; higher scores reflected poorer knowledge of viral transmission.

We assessed the knowledge of participants on 11 common symptoms and signs of COVID-19 culled from the Nigeria Centre for Disease Control (NCDC) [5]. This formed the symptom knowledge scale (Cronbach's alpha = 0.86). A maximum score of 19 was attainable and higher scores depicted better knowledge of features of the disease.

We also assessed universally acceptable measures to prevent contracting or spreading COVID-19. Questions included hand washing, use of hand sanitizers, physical distancing, healthy diet, among others. This scale was termed the preventive measures knowledge scale (Cronbach's alpha = 0.61). Correct responses had a score of 1 while incorrect responses had 0 to make a total score of 19 with regards to knowledge of preventive measures.

A previous study had assessed COVID-19 risk perception among adults in the United States using a “perceived risk perception scale” (Cronbach’s alpha = 0.72) [8]. This risk scale was validated for the study and locally adapted. Thereafter, respondents completed the validated perceived risk perception scale (Cronbach's alpha = 0.63), which had 10 survey items (five-point Likert scale: 0 = strongly disagree/disagree/neutral; 1 = agree/strongly agree). The scoring of the perceived risk perception scale, which ranges from 0 to 10, was calculated by summing the participants' responses of "Agree" and "Strongly Agree" to 10 survey items. The greater the number a participant receives on this scale, the greater their perceived risk of COVID-19 (see Appendix).

Data analysis

The Statistical Package for Social Sciences, version 22 (IBM SPSS Statistics, Armonk, NY) was used for data analysis. We reviewed and cleaned the data before the analysis. Variables were summarized using simple frequencies, proportions, and percentages. Inferential statistics (chi-square test) and bivariate analysis were used to test factors associated with acceptance of the COVID-19 vaccine. A 95% confidence interval and a significance level set at p < 0.05 were adopted.

Ethical consideration

The study was approved by the Ethical Committee of the Research and Statistics Department of Katsina State Ministry of Health, Nigeria with the study protocol code: MOH/ADM/SUB/1152/1/16. Written informed consent was obtained from each study participant before the questionnaire was administered.

## Results

**Figure 1:**
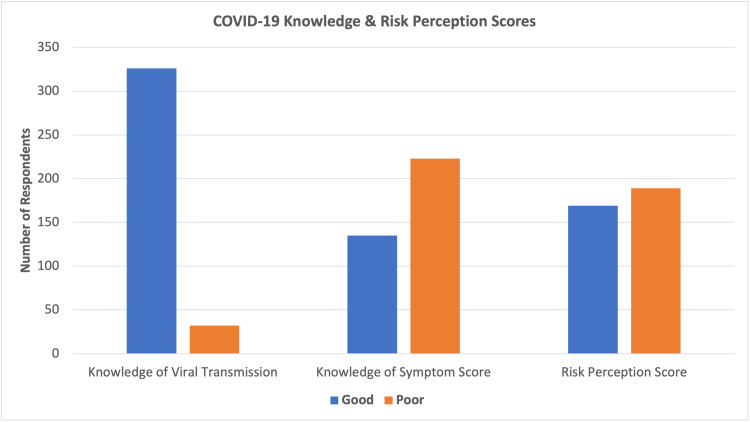
Knowledge of viral transmission, symptom score, and risk perception score.

A total of 358 out of 360 participants completed the survey, giving a completion rate of 99.4%. Table 1 shows the sociodemographic characteristics of the participants. The mean risk perception score for COVID-19 was 5.1 (±2.2 SD) with a possible maximum of 10 while the mean COVID-19 symptom knowledge score was 8.6 (±4.1 SD) with a maximum score of 19. The mean preventive measure knowledge score was 10.8 (±2.0 SD) with a maximum score of 14 (Figure 1).

**Table 1:** Sociodemographic characteristics of respondents.

Variable	Frequency	Percentage
	(N = 358)	(%)
Sex		
Male	169	47
Female	189	53
Age group (years)		
<30	171	47.8
31–40	100	27.9
41–50	50	14
51–60	27	7.5
>60	10	2.8
Tribe		
Hausa	55	15.4
Igbo	136	38.0
Yoruba	111	31.0
Others	56	15.6
Level of education		
No formal education	14	3.9
Primary	8	2.2
Junior secondary	6	1.7
Senior secondary	62	17.3
Tertiary	268	74.9
Occupation		
Unemployed/student/farmer	123	34.4
Street trader/clerks/manual laborer	32	8.9
Elementary school teacher/driver/artisan	23	6.4
Intermediate grade civil servants/high school teacher	29	8.1
Senior civil service workers, management-level professionals, entrepreneurs of large-scale companies	151	42.2
Region of residence		
West	128	35.8
East	111	31.0
North	119	33.2
Religion		
Christianity	284	78.3
Islam	73	20.4
Others	1	0.3

Of all the participants surveyed, two-thirds of them reported they would take the COVID-19 vaccine if it is recommended by healthcare workers or health agencies like WHO, NCDC, or the National Primary Healthcare Development Agency (NPHCDA). In contrast, only 71 people (19.8%) said they will take the vaccine regardless of who recommends it. About 203 (56.7%) of the participants said they will recommend the vaccine to other people.

There was a statistically significant association between the male gender and COVID-19 vaccine acceptance if recommended by a health worker (73.9%, p-value = 0.027). Similarly, we noted that participants of Hausa ethnicity were more likely to take the vaccine if recommended by a health worker compared to other ethnic groups (84.9%, p-value = 0.007). The recommendation of COVID-19 vaccines by a health worker or health agency, and the three major regions where we recruited our participants were associated with vaccine acceptance as summarized in Table 2.

**Table 2:** Association between selected demographic variables, COVID-19 vaccine acceptance, and recommending COVID-19 vaccine to others. * P-values less than 0.05 are statistically significant.

Variable	I will take the COVID-19 vaccine if recommended by a health worker or health agency		I will recommend the COVID-19 vaccine to others	
	No	Yes	No	Yes
	n (%)	n (%)	n (%)	n (%)
Sex				
Female (183)	68 (37.1)	115 (62.9)	84 (45.9)	99 (54.9)
Male (165)	43 (26.1)	122 (73.9)	61 (37)	104 (73)
	χ^2^ = 4.920	P-value = 0.027*	χ^2^ = 2.848	P-value = 0.091
Level of education				
No formal education	6 (46.2)	7 (53.8)	7 (53.8)	6 (46.2)
Primary	2 (28.6)	5 (71.4)	6 (85.7)	1 (14.3)
Junior secondary	0 (0)	6 (100)	1 (16.7)	5 (83.3)
Senior secondary	21 (35)	39 (65)	27 (45)	33 (55)
Tertiary	83 (31.6)	180 (68.4)	105 (39.9)	158 (60.1)
	χ^2^ = 4.322	P-value = 0.364	χ^2^ = 8.513	P-value = 0.074
Tribe				
Hausa	8 (15.9)	45 (84.1)	16 (30.2)	37 (69.8)
Igbo	54 (40.9)	78 (59.1)	65 (49.2)	67 (50.8)
Yoruba	32 (29.4)	77 (70.6)	50 (45.9)	59 (54.1)
Others	18 (32.7)	37 (67.3)	15 (27.3)	40 (72.7)
	χ^2^ = 12.119	P-value = 0.007*	χ^2^ = 11.454	P-value = 0.01*
Religion				
Christianity	101 (36.2)	178 (63.8)	123 (44.1)	156 (65.9)
Islam	11 (16)	58 (84)	23 (33.3)	46 (66.7)
Others	0 (0)	1 (100)	0 (0)	1 (100)
	χ^2^ = 10.892	P-value = 0.004*	χ^2^ = 3.350	P-value = 0.187
Region of residence				
West	42 (36.2)	74 (63.8)	61 (53.0)	54 (47)
East	44 (43.1)	58 (56.9)	51 (49.5)	52 (50.5)
North	20 (17.2)	96 (82.8)	30 (25.6)	87 (74.4)
	χ^2^ = 18.440	P-value = 0.001*	χ^2^ = 20.926	P-value = 0.001*

## Discussion

The proportion of people willing to take the COVID-19 vaccines from our study was similar to the 67% reported by Malik et al. at Yale but higher than 58.2% earlier reported in Nigeria by Olumofe et al. [9,16]. The figures from our study and Malik et al. were lower compared to the findings by Abdelhafiz et al. in Egypt where 73% were looking forward to getting the COVID-19 vaccine when available (this was done before the first vaccine approval) [9,18]. Since the ultimate aim of vaccination is to achieve herd immunity, the estimate from our study and the one reported by Olumofe et al. fell short of the minimum 75% vaccination rate needed to achieve herd immunity among the population. This is with the assumption that there are going to be enough vaccine doses procured to vaccinate at least 150 million Nigerians. Additionally, given the high efficacies (62-94.5%) of the vaccines approved so far, at least 75% to 95% vaccination coverage is required to achieve herd immunity [19]. Care should be taken in interpreting the estimated acceptance rate as they do not automatically translate to actual average vaccination coverage. For example, factors such as proximity and cost of going to vaccination sites and availability of vaccines could potentially affect the number of those willing to take the vaccine from getting one. Nonetheless, the data from this study can serve as an important pointer that critical stakeholders need to do more in terms of vaccine awareness and education to improve the uptake as it is being rolled out, including teasing out sensational news on perceived side effects from factual scientific evidence. A distinct picture from this study is the fact that even though an overall 66.2% of our participants indicated they would take the vaccine, many of them would do so on the recommendation of respected figures like healthcare workers or health agencies [20]. As reported by Malik et al., potential vaccine takers have the highest confidence in healthcare professionals, their physicians, and health institutions like the CDC and WHO [9]. Vaccine education offered by individuals with a better understanding of the net benefits of vaccination against SARS-Cov-2 is extremely important in combating the hesitancy that may be seen in the general Nigerian population. These tasks should not be left to healthcare providers alone. Rather, it must also come from the cross-section of religious, traditional, and political leaders who are revered and respected in society [21,22].

Educational status among the populace has been shown to affect vaccine uptake. Findings from a systematic review on the determinants of the 2009 pandemic A/H1N1 influenza vaccination were consistent with our study and similar studies done elsewhere [23,24]. The positive association between the levels of education and vaccine uptake is also consistent with polio vaccine hesitancy studies. A number of these studies showed that there were higher odds among women with no education of their child not being vaccinated for polio [25]. According to a multinational European survey on influenza vaccination uptake, the effects of education varied from one country to another [26]. In Australia and Poland, influenza vaccination was higher among educated people whereas a contrary trend was observed among educated persons in Germany and Finland [26]. This further validates the earlier suggestion that other factors could water down the influence of education on vaccine uptake.

There was a significant association between vaccine acceptance or uptake and male gender, tribe/ethnicity, religion, and place of residence. Olumofe et al. reported similar findings of the significant male influence of potential uptake of COVID-19 vaccine [16]. The influence of different levels of socio-economic statuses on the acceptance of COVID-19 vaccines in this study mirrors what others have reported both within and outside Nigeria [8,15,18,20]. As with our study, Malik et al. reported a significant association between vaccine acceptance and race/ethnicity, with being Black/African American and American Indian as independent predictors [9]. This implies that a one-size-fits-all messaging on COVID-19 and vaccines should be discouraged by health policymakers, rather, more targeted messaging should be considered.

One important improvement we did with the adaptation of the study by Malik et al. was the field approach we used in collecting data. By default, online surveys will naturally exclude people who do not have access to the internet, which is more than half of the Nigerian population as the estimated internet penetration in Nigeria is 46.6% [27]. This potential source of selection bias was eliminated in our study. On the other hand, a multistage stratified sampling technique was used for this population as described in the methodology. However, the stratification to an extent led to limitations in the inclusion of some formal groups of the general population. Additionally, due to funding restraints, the lack of stratification to the recognized geopolitical zones in Nigeria likely introduced some bias. For example, about 91% of our participants had at least a secondary school education, which is not in keeping with a lower adult literacy level in Nigeria. While we made efforts to avoid bias, as outlined in our sampling methodology, given our limited resources, there was still some degree of bias in the results obtained, thus affecting its representativeness of the general population.

## Conclusions

Over 60% of Nigerians are willing to take the COVID-19 vaccine if recommended by a healthcare worker. Our study has highlighted important considerations and potential targets to incorporate in improving vaccine uptake. More than 50% of the respondents had a poor risk perception of being infected by COVID-19. Notably, being male, following the Islamic religion, being of the Hausa tribe, and living in the northern part of the country all have a significant positive association with COVID-19 vaccine uptake. More women, especially in southern Nigeria, need to be targeted, in addition to engaging influential religious and traditional leaders in vaccination messaging. Besides the insufficient supply of vaccines, vaccine hesitancy remains a major limitation to optimum vaccination coverage. It is therefore pertinent that as the supply of COVID-19 vaccines increasingly becomes available to Nigeria, strategies that are centered around support for public health agencies and health workers are key for effective public health messaging to increase uptake of the vaccines.

## Appendices

Questionnaire

A. Sociodemographics

1. Gender

o Male

o Female

2. Age ____________ (years)

3. Education

o No formal education

o Primary school

o Junior secondary

o Senior secondary/Nigeria Certificate in Education/other professional training

o Tertiary

4. Tribe

o Hausa

o Igbo

o Yoruba

o Others ______________

5. Occupation

o Unemployed/full housewife/student/subsistence farmers

o Petty trader/messengers/laborers

o Junior school teacher/driver/artisan

o Intermediate grade civil servant/senior school teacher

o Senior civil servant/professionals/managers/large scale traders/businessman/contractor

6. Religion

o Christianity

o Islam

o Others

7. State of residence ___________

B. Medical History

8. Do you have any known chronic medical conditions such as

o Hypertension (high blood pressure)

o Diabetes mellitus

o Asthma

o Peptic ulcer

o Chronic obstructive pulmonary disease

o Others _____

9. Have you had any respiratory tract infections in the last five months

o Yes

o No

10. Do you think you might have been sick with COVID-19 in the last five months?

o Yes (confirmed with a test)

o Yes (but not confirmed with a test)

o No, I have never been infected with COVID-19

o I am not sure

11. Do you know in your immediate social network anyone sick with novel COVID-19?

o Yes, confirmed

o Yes, but not yet confirmed

o No

o I am not sure

C. Knowledge of COVID-19

Instructions: The following are questions that will ask about your knowledge level on COVID-19. Please select the answer of your choice.

1. Are you aware of the novel coronavirus/COVID-19 pandemic?

o Yes

o No

o Don’t know

2. How did you learn about the novel coronavirus/COVID-19pandemic?

o Television

o Newspapers/magazines

o Websites

o Family/friends

o Healthcare professionals (e.g., doctors, nurses, paramedics, and pharmacists)

o Health officials (e.g., Centers for Disease Control and Prevention and National Institute of Health)

o Social media

3. How would you rate your knowledge level on novel coronavirus/COVID-19?

o Very poor

o Poor

o Average

o Good

o Very good

4. Which of the following is correct about the definition of novel coronavirus/COVID-19?

o Novel coronavirus/COVID-19 is a respiratory disease caused by a viral infection

o Displayed symptoms usually include respiratory symptoms accompanied by fever, but novel coronavirus/COVID-19 is not contagious

o Novel coronavirus/COVID-19 can progress to a severe illness but never leads to death

o Don’t know

5. Which of the following is correct about the transmission route of novel coronavirus/COVID-19?

o Novel coronavirus/COVID-19 is transmitted through coughing or sneezing

o Novel coronavirus/COVID-19 is not transmitted by close contact with people

o Don’t know

6. Which of the following is correct about “close contact” of novel coronavirus/COVID-19?

o “Close contact” involves direct contact with persons’ respiratory secretions

o Relatives and healthcare workers are excluded from the category of close contact

o Don’t know

D. COVID-19 and Vaccination

1. Which one is correct about the available treatment or vaccine for COVID-19?

a. There is a curative treatment for novel coronavirus/COVID-19 (the question below pops up if this option is selected)

i. If you believe/think that there is a curative treatment for COVID-19, how did you hear about it?

o Television

o Newspapers/magazines

o Websites

o Family/friends

o Healthcare professionals (e.g., doctors, nurses, paramedics, and pharmacists)

o Health officials (e.g., Centers for Disease Control and Prevention and National Institute of Health)

o Social media

o Don’t know

b. Currently, there is neither a curative treatment nor a vaccine

c. Currently, there isn’t a curative treatment, but there is a vaccine

i. How did you learn about the vaccine?

o Television

o Newspapers/magazines

o Websites

o Family/friends

o Healthcare professionals (e.g., doctors, nurses, paramedics, and pharmacists)

o Health officials (e.g., Centers for Disease Control and Prevention and National Institute for Health)

o Social media

o Don’t know

**Table 3:** Personal attitude to COVID-19 and vaccine. NCDC, Nigeria Centre for Disease Control.

	Strongly disagree	Disagree	Neutral	Agree	Strongly agree	Don’t know
The vaccine will be effective against COVID-19						
Drug companies are working to make the COVID-19 vaccine available						
COVID-19 vaccines are already available in some parts of the world but not yet in Nigeria						
If a vaccine becomes available, I will take it only if it is recommended for me by health workers or agencies like WHO and NCDC						
If a vaccine becomes available I will take it, recommended or not						
Will you recommend your family members, friends, or associates to take the COVID-19 vaccine if it becomes available						
I cannot take or advise anyone I know to take the COVID-19 vaccine if it becomes available						

**Table 4:** Which of the following are effective preventative measures for yourself and/or others against COVID-19?

	Yes	No	Don’t know
Hand washing			
Avoiding touching your eyes, nose, and mouth with unwashed hands			
Using disinfectants			
Staying home when you are sick			
Taking herbal supplements			
Covering your cough or sneeze			
Eating a balanced diet			
Avoiding close contact with someone who is sick			
Using caution when opening mail			
Avoiding eating meat			
Exercising regularly			
Wearing a face mask			
Using hand sanitizer			
Social/physical distancing			

**Table 5:** Which of the following are symptoms of COVID-19?

	Yes	No	Don’t know
Fever			
Dry cough			
Shortness of breath			
Sore throat			
Runny or stuffy nose			
Muscle or body aches			
Headaches			
Fatigue (tiredness)			
Vomiting or diarrhea			
Chills			
Ear pain			
Sinus pain			
Chest pain			
Wheezing			
Just any form of un-wellness			

**Table 6:** Risk perception on COVID-19.

		Strongly disagree	Disagree	Neutral	Agree	Strongly agree	Don’t know
1	My health will be severely damaged if I contract COVID-19.	1	2	3	4	5	6
2	I think COVID-19 is more severe than an ordinary cold.	1	2	3	4	5	6
3	Even if I fall ill with another disease, I will not go to the hospital because of the risk of getting COVID-19 in the hospital.	1	2	3	4	5	6
4	COVID-19 will inflict serious damage in my community.	1	2	3	4	5	6
5	COVID-19 will spread widely in Nigeria.	1	2	3	4	5	6
6	I am more likely to get COVID-19 than other people.	1	2	3	4	5	6
7	I believe I can protect myself against COVID-19.	1	2	3	4	5	6
8	I believe I can protect myself against COVID-19 better than other people.	1	2	3	4	5	6
9	I am scared about COVID-19.	1	2	3	4	5	6
10	COVID-19 has severely damaged the economy.	1	2	3	4	5	6
11	The federal government has effectively controlled COVID-19.	1	2	3	4	5	6
12	My state government has effectively controlled COVID-19.	1	2	3	4	5	6
13	The number of cases of COVID-19 will increase in Nigeria over the next month.	1	2	3	4	5	6
14	I think social distancing is effective at slowing the spread of the pandemic.	1	2	3	4	5	6
15	Slowing the spread of the pandemic is more important than damage to the economy.	1	2	3	4	5	6
16	Public health should be prioritized over opening the economy.	1	2	3	4	5	6
17	Saving lives is more important than personal liberty.	1	2	3	4	5	6
19	For me, avoiding an infection with the COVID-19 in the current situation is easy.	1	2	3	4	5	6
20	COVID-19 makes me feel helpless.	1	2	3	4	5	6
22	Community facilities such as schools or kindergartens should be closed.	1	2	3	4	5	6
23	Major events should be canceled by the organizers.	1	2	3	4	5	6
24	I worry about the health system being overloaded by people sick with COVID-19.	1	2	3	4	5	6
27	I think social distancing measures should be extended until public health authorities say it is safe to stop.	1	2	3	4	5	6

## References

[1] Bowale A, Abayomi A, Idris J (2020). Clinical presentation, case management and outcomes for the first 32 COVID-19 patients in Nigeria. Pan Afr Med J.

[2] Egbu E, Ihemedu C, Egbu AC (2020). Multisystemic manifestations of COVID-19 in a 37 year old Nigerian man: a case report. Int J Ophthalmol Clin Res.

[3] Garg M, Al-Ani A, Mitchell H, Hendy P, Christensen B (2020). Editorial: low population mortality from COVID-19 in countries south of latitude 35 degrees North—supports vitamin D as a factor determining severity. Authors' reply. Aliment Pharmacol Ther.

[4] Suleiman AA, Suleiman A, Abdullahi UA, Suleiman SA (2021). Estimation of the case fatality rate of COVID-19 epidemiological data in Nigeria using statistical regression analysis. Biosaf Health.

[5] (2021). Nigeria Centre for Disease Control. COVID-19 Nigeria. NCDC. COVID-19 Nigeria.

[6] (2021). Johns Hopkins University. Nigeria vaccine tracker. https://coronavirus.jhu.edu/region/nigeria.

[7] Onapajo H, Adabiyi J (2020). 'COVID-19 is big scam': citizens distrust and the challenges of combating coronavirus in Nigeria. https://republic.com.ng/february-march-2020/distrust-nigeria-coronavirus/.

[8] (2020). World Health Organization. COVID-19 vaccines. Vaccines.

[9] Malik AA, McFadden SM, Elharake J, Omer SB (2020). Determinants of COVID-19 vaccine acceptance in the US. EClinicalMedicine.

[10] COCONEL Group (2020). A future vaccination campaign against COVID-19 at risk of vaccine hesitancy and politicisation. Lancet Infect Dis.

[11] Shetty P (2010). Experts concerned about vaccination backlash. Lancet.

[12] Larson HJ, de Figueiredo A, Xiahong Z (2016). The state of vaccine confidence 2016: global insights through a 67-country survey. EBioMedicine.

[13] Neia Prata Menzes, Muloongo Simuzingili, Zelalem Yilma Debebe, Fedja Pivodic Ernest Massiah (2021). What is driving COVID-19 vaccine hesitancy in Sub-Saharan Africa?. https://blogs.worldbank.org/africacan/what-driving-covid-19-vaccine-hesitancy-sub-saharan-africa.

[14] (2021). The share Of U.S. adults willing to get vaccinated ticks up, a new poll finds. https://www.npr.org/2021/09/03/1033750072/the-share-of-u-s-adults-willing-to-get-vaccinated-ticks-up-a-new-poll-finds.

[15] Gyang D. Facebook2020 (2020). Davou Gyang interview. https://www.facebook.com/jatau.davougyang/videos/1311362892391255/.

[16] Olumofe CO, Soyemi K, Udomah BF (2021). Predictors of uptake of a potential Covid-19 vaccine among Nigerian adults. J Vaccines.

[17] (2021). The world factbook: Nigeria. https://www.cia.gov/the-world-factbook/countries/nigeria/.

[18] Dean AG, Sullivan KM, Soe MM (2020). OpenEpi: open source epidemiologic statistics for public health, version 2020. http://www.OpenEpi.com.

[19] Abdelhafiz AS, Mohammed Z, Ibrahim ME, Ziady HH, Alorabi M, Ayyad M, Sultan EA (2020). Knowledge, perceptions, and attitude of Egyptians towards the novel coronavirus disease (COVID-19). J Community Health.

[20] Anderson RM, Vegvari C, Truscott J, Collyer BS (2020). Challenges in creating herd immunity to SARS-CoV-2 infection by mass vaccination. Lancet.

[21] Fu C, Wei Z, Pei S, Li S, Sun X, Liu P (2020). Acceptance and preference for COVID-19 vaccination in health-care workers (HCWs). medRxiv.

[22] Liu Q, Luo D, Haase JE (2020). The experiences of health-care providers during the COVID-19 crisis in China: a qualitative study. Lancet Glob Health.

[23] Kwok KO, Li KK, Wei WI, Tang A, Wong SYS, Lee SS (2021). Editor's choice: influenza vaccine uptake, COVID-19 vaccination intention and vaccine hesitancy among nurses: a survey. Int J Nurs Stud.

[24] Brien S, Kwong JC, Buckeridge DL (2012). The determinants of 2009 pandemic A/H1N1 influenza vaccination: a systematic review. Vaccine.

[25] Khan MT, Zaheer S, Shafique K (2017). Maternal education, empowerment, economic status and child polio vaccination uptake in Pakistan: a population based cross sectional study. BMJ Open.

[26] Endrich MM, Blank PR, Szucs TD (2009). Influenza vaccination uptake and socioeconomic determinants in 11 European countries. Vaccine.

[27] (2021). Internet user penetration in Nigeria from 2016 to 2026. https://www.statista.com/statistics/484918/internet-user-reach-nigeria/.

